# Environmental Barriers to Participation of Preschool Children with and without Physical Disabilities

**DOI:** 10.3390/ijerph14050518

**Published:** 2017-05-11

**Authors:** Lin-Ju Kang, Ming-Chieh Hsieh, Hua-Fang Liao, Ai-Wen Hwang

**Affiliations:** 1 Graduate Institute of Early Intervention, College of Medicine, Chang Gung University, 259 Wen-Hwa 1st Road, Kwei-Shan, Tao-Yuan City 333, Taiwan; linjukang@mail.cgu.edu.tw (L.-J.K.); donaldduckmigi@gmail.com (M.-C.H.); 2 Department of Physical Medicine and Rehabilitation, Chang Gung Memorial Hospital, Linkou, 5 Fu-Xing St., Kwei-Shan, Tao-Yuan City 333, Taiwan; 3 School and Graduate Institute of Physical Therapy, College of Medicine, National Taiwan University, 17 Xuzhou Road, Taipei City 100, Taiwan; hfliao@ntu.edu.tw

**Keywords:** environment, home, school, community, barriers, physical disabilities, preschool children

## Abstract

Environment plays a vital role in affecting participation of young children in home, school, and community. Knowledge of environmental barriers helps to develop solutions or strategies that enable participation. The study compared the environmental barriers perceived by parents of preschool children with physical disabilities (PD, *n* = 142) and with typical development (TD, *n* = 192) in Taiwan. Parents identified environmental barriers by structured interview using the Chinese version of the Child and Adolescent Scale of Environment (CASE-C). The CASE-C is an 18-item measure of the impact of problems with physical, social, and attitudinal environmental features. Differences between the PD and TD groups in the summary scores for the CASE-C and the percentages of parents who perceived a problem for each item were examined by the analysis of covariance (ANCOVA) and Chi-square test. Parents of children with PD more often identified barriers related to family resources and community programs or services, social attitudes, assistance and supports outside of home, physical design of home and community, transportation, and assistive devices or equipment. Greater impacts of barriers were also reported by parents of preschool children with PD. Our findings provide evidence of environmental barriers that inform practice and policies to modify the barriers and provide an accessible and inclusive environment for families with young children.

## 1. Introduction

Environmental factors play a vital role in either supporting or hindering participation of young children in home, preschool, and community contexts [1,2,3]. Environmental factors refer to the physical, social, and attitudinal features that surround a child, as defined in the International Classification of Functioning, Health, and Disability (ICF) [4]. The five domains of environmental factors framed by the ICF include “Products and technology”, “Natural and human-made changes”, “Support and relationship”, “Attitude”, and “Services, systems and policies” [4]. For young children who have or are at risk for developmental disabilities, the Division for Early Childhood (DEC) Recommended Practices (2014) suggest aspects of physical (e.g., space and equipment), social (e.g., attitude and relationship of peers and other family members), and temporal (e.g., sequence of routines and activities) environments that can be altered to support young children’s learning [5]. Practitioners and families are encouraged to provide inclusive and enriched learning environments that fosters children’s overall health and development [5].

Children who have health conditions associated with physical disabilities (PD), such as cerebral palsy, or motor or developmental delay, have impairments in body functions and activity limitations that often restrict their daily participation. However, unsupportive environments can even have greater impacts on the restriction of participation [2,6]. Previous research has focused on school-age children with disabilities, in which inaccessible physical environments, negative societal attitudes, and lack of supports, assistance, and resources were frequently identified barriers to participation [7,8,9,10,11,12]. Among the few studies involving preschool children with disabilities, lack of time and money, limited access to social support, transportation, programs, and services were perceived as barriers to preschool or community participation [1,13]. Despite the growing evidence on the environmental impacts on children’s participation, little is known about environmental barriers experienced by preschool children with disabilities in Taiwan. Knowledge of environmental barriers will help to develop solutions or strategies of environmental modification to support children’s access to and participation in daily activities [5,13,14,15].

Research indicates diverse patterns of home, education, or community environmental supports/barriers to participation between children with and without disabilities [16,17,18]. To be specific, parents of school-age children with disabilities tend to report more environmental barriers and less supports in home, school, and community than those of children without disabilities [19,20,21]. On the other hand, parents of children without disabilities were more likely to consider an environmental factor to not be an issue, or indicated no additional needs for resources such as public transportation and community programs or services [20,21]. Similarly, parents of preschool children with developmental disabilities reported less preschool and community environmental supports in comparison to parents of children without disabilities [1,3]. However, it is still not clear whether parent-reported barriers differ between children with and without disabilities [1]. To our knowledge, no study has used a comprehensive environment measure to capture the barriers from home, school, and community settings for preschool children with and without disabilities in Taiwan.

The Child and Adolescent Scale of Environment (CASE) is designed to measure environmental features that impact children’s participation across home, school, and community settings [22]. The CASE has been successfully used in several studies involving children with acquired brain injury and a variety of chronic conditions [12,22,23]. Previously, we reported evidence of cross-cultural validation of the Chinese version of the CASE (CASE-C) among Taiwanese children 6–18 years with a variety of disabilities [23]. The impact of environmental barriers was found to vary on the basis of severity of impairments and medical conditions of children. However, we did not include children under the age of 6 or a comparison group of children without disabilities. This present study aimed to identify the environmental barriers perceived by parents of preschool children with and without physical disabilities in Taiwan. There were two research questions:

1. What environmental factors were perceived by parents as barriers to participation of children with and without physical disabilities?
2. Does the impact of environmental barriers on participation differ between children with and without physical disabilities?

## 2. Methods

### 2.1. Participants

Participants were a convenience sample of 142 parents of children with PD and 192 children with typical development (TD). Participants were recruited from 11 out of 20 cities/counties in Taiwan. The participants are part of an ongoing prospective study which aims to identify the child, family, and environmental determinants of change in participation of preschool children with and without physical disabilities [24]. In the larger study, sample size was estimated based on the proposed regression models on participation that involved 10 potential predicting variables. Based on the principle that *N* = 50 + 8 (*m*), where *m* is the number of predicting variables (i.e., *m* = 10) [25], a minimum of 130 participants was required for each group. Our sample exceeded the minimum required number and thus could provide sufficient power and allow the possibility of attrition during the follow-ups. For children with PD, children were included if they were 2 to 6 years of age and had a primary medical diagnosis or condition associated with a physical disability, including cerebral palsy, acquired brain injury, developmental delay, spina bifida, congenital limb deformity, or deficiencies, etc. Children were excluded if they had associated psychological and mental health conditions that might influence participation, such as attention deficit disorder, autism spectrum disorder, clinical depression, or other emotional disorders. Medical conditions were determined by children’s physicians and reported by their parents. Children with TD were included if they were 2 to 6 years of age and did not have conditions associated with developmental disabilities or delays. Ethical approval was provided by the Institutional Review Board of Chang Gung Memorial Hospital in Taiwan (No. 103-6558C, Date of approval: 22 January 2015). Informed consent was provided by parents of all children.

Demographic information for the children and their families are summarized in Table 1. Children with PD involved 92 boys (65%) with a mean age of 4.09 years (Standard deviation (SD) = 1.30). The primary medical conditions reported by parents were primarily cerebral palsy (37%) and developmental delay (37%) (Table 2). Because of the young age and uncertainty in developmental progress, preschool children might have a transient diagnosis of “developmental delay” before the formal diagnosis related to PD. About 76% children with PD were ambulatory either with or without assistive devices (Table 2), thus representing a sample with relatively high physical functioning. Children with TD involved 109 boys (57%) with a mean age of 3.86 (SD = 1.16). More parents of children with TD were employed and reported higher levels of education and household income (*p* < 0.001).

### 2.2. Measure and Procedures

**Figure 1:**
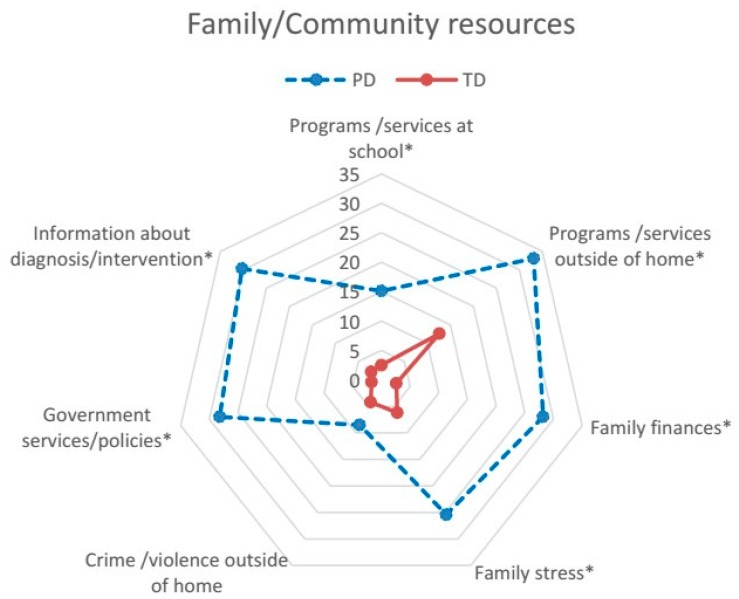
Percentage of parents who perceived barriers in each item of the subscale of Family/Community resources in the PD and TD groups. Abbreviation: PD, physical disabilities; TD, typical development; NOTE: Bonferroni adjustment of the significance level set at * *p* < 0.002.

**Figure 2:**
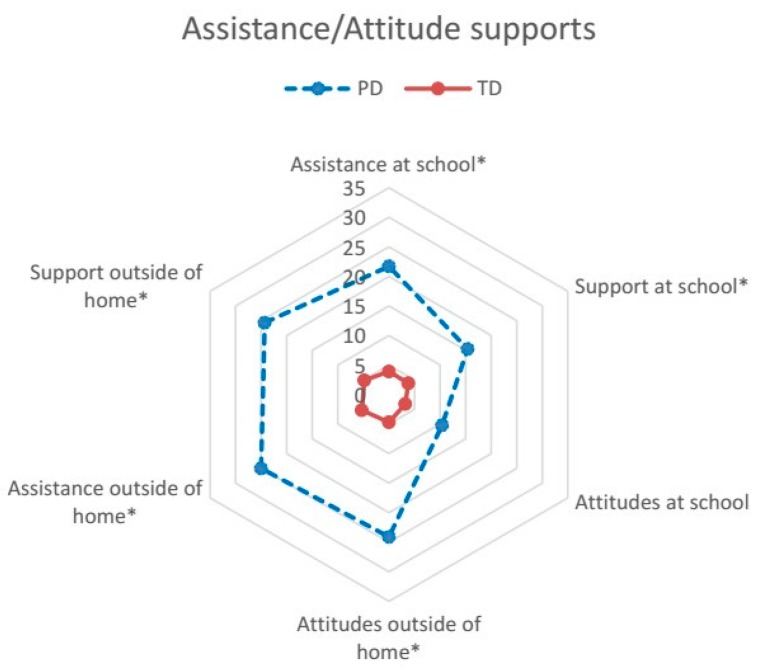
Percentage of parents who perceived barriers in each item of the subscale of Assistance/Attitude supports in the PD and TD groups. Abbreviation: PD, physical disabilities; TD, typical development. NOTE: Bonferroni adjustment of the significance level set at * *p* < 0.002.

**Figure 3:**
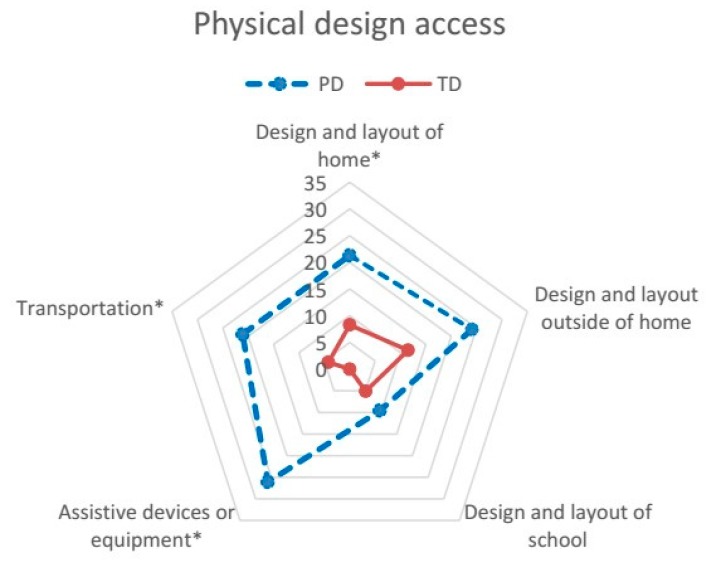
Percentage of parents who perceived barriers in each item of the subscale of Physical design access in the PD and TD groups. Abbreviation: PD, physical disabilities; TD, typical development. NOTE: Bonferroni adjustment of the significance level set at * *p* < 0.002.

The CASE-C, as the original version [26], is an 18-item measure of the impact of physical, social, and attitudinal environment on participation in daily activities, and problems related to supports, assistance, or resources in home, school, and community. The items pertaining to school apply to some type of structured school or program (e.g., preschool, day care) the young child attends [26]. The items for the CASE-C have been presented in our previous publication [23] and shorted items are shown in Figure 1, Figure 2 and Figure 3. Each item is rated on a 3-point scale: “no problem (1)”, “little problem (2)”, and “big problem (3)”; and “not applicable”. For example, if the child did not attend any type of preschool or day care, the parent may indicate “not applicable” to items pertaining to school. Following the standard scoring guidelines [26], the CASE-C total and subscale score was calculated as the sum of all applicable items divided by the maximum possible score of applicable items, and then multiplied by 100 to conform to a scale score ranging from 33.33 to 100. Higher scores indicate a greater extent of environmental problem. The scores were calculated for the total (all items) and the three subscales: Family/Community resources (seven items), Assistance/Attitude supports (six items), and Physical design access (five items) [23]. The CASE-C total and subscale scores have been reported to have adequate internal consistency (Cronbach’s α = 0.74–0.86), test-retest reliability (Intra-class correlation coefficient (ICC) = 0.73–0.90), convergent and discriminant validity [23]. For item-level analysis, the percentage of parents who indicated each environmental factor as a problem (merging the answers of “little problem” and “big problem”) was calculated for each group.

Parents who agreed to participate in the study first answered a set of child and family demographic questions, and then completed the CASE-C by structured interview with a research assistant. During this process, the assistant might have responded to parents’ questions and clarified the wording of items, if needed.

### 2.3. Data Analysis

Data screening revealed a completed dataset of the CASE-C. Data of applicable items were included in the subsequent analyses based on the scoring guidelines of the CASE [26]. Descriptive statistics were used to present the total and the three subscale scores of the PD and TD groups. For item-level analysis, the percentage of parents who perceived barriers in each group was graphed using radar plots to enable visual presentation of the data [27]. To compare the impact of environmental barriers to participation between the PD and TD groups, analysis of covariance (ANCOVA) was used to examine the differences in the CASE-C scores between groups. The parental education, employment, and household income categories were entered as covariates because group differences in these variables were found in our sample. Partial eta squares (η*_p_*^2^) were calculated to examine effect sizes (ES) between the two groups according to Kirk’s classification (small effect = 0.01–0.05; medium effect = 0.06–0.13; large effect ≥ 0.14) [28]. To reduce the chance of Type I error due to multiple comparisons, significance level for ANCOVA was set at *p* < 0.0125 after Bonferroni corrections (i.e., 0.05/4). For item-level comparisons, the Chi-square test was used to examine the differences in the percentage of parents who perceived barriers in the PD and TD groups. Significance level for the Chi-square test was set at *p* < 0.002 after Bonferroni corrections (i.e., 0.05/18) due to multiple comparisons. Previous studies [1,19,21] have informed the analytical approaches used in this study.

## 3. Results

### 3.1. The Identification of Environmental Barriers in Two Groups

The mean total and subscale score of the CASE-C are reported in Table 3. The mean scores of the TD group ranged from 34.72 to 35.50 and near the minimum scores of 33.3 (“no problem”). The mean scores of the PD groups ranged from 40.77 to 43.47 and between “no problem” to “little problem”.

The percentages of parents who perceived problems in the 18 items for the CASE-C are presented in Figure 1, Figure 2 and Figure 3. For the TD group, the percentages of parents for most items are less than or equal to 5% except Programs/services outside of home (13%), Design and layout outside of home (12%), Design and layout of home (8%), and Family stress (6%). For the PD group, the percentages of parents for most items are 15% and above, except Crime/violence outside of home (9%), Attitudes at school (10%), and Design and layout of school (10%).

### 3.2. Comparison of Environmental Barriers between Two Groups

After controlling for parental education, employment, and income, significant differences between two groups were found for the CASE-C total scores. Parents of children with PD perceived higher impacts of environmental barriers than parents of children with TD with a large effect (*η_p_*^2^ = 0.23, *p* < 0.001). The group differences were also found for the three subscale scores with medium to large effects (*η_p_*^2^ = 0.11–0.20, *p* < 0.001).

Among the seven items of the Family/Community resources pertaining to family resources and community programs or services barriers, significant differences between two groups were found for six items (i.e., except Crime/violence outside of home) (Figure 1). A higher percentage of parents from the PD group, compared with those of the TD group, indicated that they had insufficient programs or services provided by school (15% versus 3%, *χ*^2^ = 14.60, *p* < 0.001), outside of home (33% versus 13%, *χ*^2^ = 20.07, *p <* 0.001), and government agencies (28% versus 2%, *χ*^2^ = 47.07, *p* < 0.001), and needed more information about their child’s diagnosis and intervention (30% versus 2%, *χ*^2^ = 51.02, *p* < 0.001). In addition, higher percentage of parents from the PD group perceived problems with family finances (28% versus 3%, *χ*^2^ = 45.76, *p* < 0.001) and family stress (25% versus 6%, *χ*^2^ = 24.20, *p* < 0.001).

Significant group differences were found for five items pertaining to attitudes, assistance, and supports from others (i.e., except Attitudes at school) (Figure 2). A higher percentage of parents from the PD group perceived problems with attitudes from community members (24% versus 5%, *χ*^2^ = 27.28, *p* < 0.001), and indicated that they had inadequate assistance (25% versus 5%, *χ*^2^ = 26.46, *p* < 0.001) and supports (24% versus 5%, *χ*^2^ = 26.98, *p* < 0.001) outside of home, as well as inadequate assistance (22% versus 4%, *χ*^2^ = 20.74, *p* < 0.001) and supports (15% versus 4%,*χ*^2^ = 11.22, *p* = 0.001) at school than those of children with TD.

For barriers related to physical design and access, there were significant differences between two groups in four items (Figure 3). A higher percentage of parents from the PD group indicated that they had insufficient transportation (21% versus 4%, *χ*^2^ = 23.29, *p* < 0.001) and needed assistive devices or equipment (26% versus 0%, *χ*^2^ = 44.69, *p* < 0.001) than those of the TD group. Significant differences for Design and layout of home (21% versus 8%, *χ*^2^ = 11.44, *p* = 0.001) and borderline significance for Design and layout outside of home (24% versus 12%, *χ*^2^ = 9.31, *p* = 0.002) were also found between groups.

## 4. Discussion

Parents of preschool children with PD identified more environmental barriers and perceived higher impacts of barriers than parents of children with TD. The results were comparable to previous studies involving young children in the United States [3,16] and Singapore [1] and expand our current knowledge about environmental challenges of young children in Taiwan. Based on the biopsychosocial model of the ICF, disability is the result of interactions between health conditions and contextual factors (e.g., environmental factors) [4]. Developmental and health assessment is recommended to evaluate the environmental factors as well as other components of the ICF to get a comprehensive functional profile for each individual with developmental needs [29]. In Taiwan, measurement of children’s participation and environmental factors is a key component of the assessment in the new Disability Evaluation System (DES) [30,31]. The national sample demonstrated that children and youth with disabilities who had higher levels of severity of impairment encountered more environmental barriers and those experiencing more environmental problems also had greater restrictions in participation [32]. This present study further identified that children with PD and TD experienced different patterns of environmental barriers, which supports the interplay of child’s functioning and surrounding environment within the ICF. Therefore, environmental factor assessment is very important for children with disabilities.

Despite the group differences, the results were encouraging, given that the scores for both groups indicated a relatively low impact of environmental barriers (i.e., scores were close to the minimum scores of 33.3). The ratings may suggest that efforts have been made to create an available and accessible environment that supports participation of young children. The results may also be explained by the young age of our sample. Living environment and daily activities performed by young children are more easily arranged and managed by parents than that of older children. It is possible that parents may perceive more environmental problems as their children grow and expand living areas.

Parents of children with PD more often identified needs for improving availability and adequacy of information, and community and governmental resources. Parents of children with PD in our study frequently indicated needs for information about intervention resources, and wanted adapted community programs for their child [33]. In addition, parents of children with PD were more often worried about financial issues and also reported problems with family stress that might hinder their child’s participation. In our sample, there were more children with PD whose parent respondents were not employed and lived in households with lower income levels. During the interviews in our study, some parents of children with PD indicated having financial burden after resigning from jobs to take care of their children [33]. The above factors seemed less of an issue for children with TD, as a relatively low percentage of parents (2–13%) perceived problems. A relatively prominent barrier is programs and services outside of home (identified by 13% parents of children with TD).

Parents of children with PD more often perceived problems with assistance, attitudes, and supports from others in places outside of home. For example, some parents of children with PD in our study reported that they felt uncomfortable by the ways people looked at them and their children while going out. Parents also reported lack of assistance from service providers when they took a taxi or went to a hospital [33]. Negative social attitudes have been identified as a key barrier that might even prevent change in other aspects of barriers, such as problems with physical environment [34,35]. Parents of children with PD also indicated needs for additional help and supports at preschool, which reflects the limited time and manpower of school personnel. Preschool educators often spend a lot of time in managing the whole class and may not have either the time or ability to address the special needs of each child [36,37]. Professional supports are needed to enable children’s engagement in classroom activities [36,38].

Parents of children with PD were more often worried about the inaccessible physical environments at home and nearby areas, and lack of transportation systems and assistive devices or equipment, which were frequently mentioned barriers in the literature [34,39]. We also noticed an increased percentage of parents of children with TD who perceived problems with design and layout at home and nearby areas. For example, parents may want more spaces for their child to move around and play safely, and need public toilets or buses to be appropriate heights. Those were common problems encountered by parents of young children regardless of disability. This highlights that some environmental barriers are common for children with and without disabilities and that environmental accessibility is important for all children.

Children have the right to live in an environment that enables resources provision, protection, and participation [40]. A universal design [41] and developmental appropriate approach [42] suggest eliminating barriers to create an inclusive and rich learning environment. Our findings have provided evidence of environmental barriers that inform child care practices and policies to form a supportive environment for families with young children. The CASE-C measures physical and social environmental features and resources which are aligned with the ICF and DEC Recommended Practices. The CASE-C provides a comprehensive measure for practitioners and parents to identify barriers and plan strategies collaboratively to modify and adapt the environment. For example, practitioners may discuss family financial situations and stress with parents, and provide information or resources based on family needs. Practitioners may provide emotional supports to families who perceive negative social attitudes, and advocate promoting acceptance of community members. Positive attitudes and assistance from others to children and parents in need can be encouraged by providing education and promotion of the value of inclusion in the community and society.

Regulations in Taiwan, such as the *People with Disabilities Rights Protection Act* amended in 2015 [43], promote applying the concept of universal design to the development of public facilities, product and technology, transportation systems, and services. Local authorities have the responsibility to provide social, cultural, recreation, and leisure activities to optimize community inclusion of individuals with disabilities. However, what we learned from this study is that parents might not know their rights or currently available resources, and thus many parents did not get enough of the resources they need. It is important for local governments to fully implement social welfare policies and establish an integrated and accessible information system for young children with disabilities.

There are several limitations of this study which inform future research directions. This is a sample of convenience with relatively high physical functioning, and therefore the sample considered in this study might not be representative of all young children with and without PD in Taiwan. During recruitment, it was not feasible to individually match the two groups by age, income, or other factors known to influence participation. In addition, environmental barriers may vary depending on various factors such as children’s functional ability and living areas [39]. Further study on a large and representative sample of Taiwanese children would enable the investigation of variation in environmental impacts between children with different abilities who reside in different regions. This study did not measure positive environmental features that promote participation and thus this warrants further investigation. As part of a larger study, this study focused on identifying environmental barriers reported by the parents but did not, as of yet, examine their associations with children’s actual participation. Previous studies suggest a medicating role of environmental features between the child and family factors and participation of children with and without disabilities [2,6]. Further analyses of our study will focus on the influence of child, family, and environmental factors collectively on participation of preschool children with and without PD.

## 5. Conclusions

This is the first study, to our knowledge, that provides a comprehensive comparison of patterns of environmental barriers between preschool children with and without disabilities in Taiwan. Parents of children with PD more often identified barriers related to limited access to family resources and community programs or services, lack of positive social attitudes, assistance, and supports outside of home, inaccessible physical environment, and inadequacy of transportation and assistive devices or equipment. Parents of children with PD also perceived higher impacts of environmental challenges than parents of children with TD. Environmental barriers identified in this study will inform childhood health research, practice, and policies to change environmental demands and optimize participation.

## Figures and Tables

**Table 1:** Child and family demographic information.

Variables	PD (*n* = 142)	TD (*n* = 192)	*χ*^2^	*p*-Value
Child’s age			3.48	0.062
< 4 years	72 (50.7%)	117 (60.9%)		
≥ 4 years	70 (49.3%)	75 (39.1%)		
Child’s sex			2.03	0.154
Boys	92 (64.8%)	109 (57.1%)		
Girls	50 (35.2%)	82 (42.9%)		
Child schooling			0.11	0.743
School	105 (73.9%)	145 (75.5%)		
At home	37 (26.1%)	47 (24.5%)		
Parent respondents, *n* (%)			1.93	0.381
Mother	120 (84.5%)	171 (89.1%)		
Father	21 (14.8%)	19 (9.9%)		
Grandparent	1 (0.7%)	2 (1.0%)		
Parental education			43.47	<0.001
Junior high school and under	9 (6.3%)	0		
High school graduate	47 (33.1%)	26 (13.5%)		
College/university graduate	71 (50.0%)	107 (55.7%)		
Graduate degree	13 (9.2%)	57 (29.7%)		
Unanswered	2 (1.4%)	2 (1.0%)		
Parental employment status			19.64	<0.001
Employed	65 (45.8%)	134 (69.8%)		
Unemployed	75 (52.8%)	56 (29.2%)		
Unanswered	2 (1.4%)	2 (1.0%)		
Household income ^a^			38.14	<0.001
<$800,000	89 (62.7%)	73 (38.0%)		
$800,000–$1,800,000	44 (31.0%)	95 (49.5%)		
>$1,800,000	4 (2.8%)	23 (12.0%)		
Unanswered	5 (3.5%)	1 (0.5%)		

^a^ Unit: New Taiwan Dollars (NTD$30 = USD$1) Abbreviation: PD, physical disabilities; TD, typical development.

**Table 2:** Primary conditions and methods of mobility of children with physical disabilities (*n =* 142).

Variables	*n* (%)
Primary conditions	
Cerebral palsy	53 (37.3%)
Developmental delay	53 (37.3%)
Chromosomal disorder	20 (14.1%)
Acquired brain injury ^a^	13 (9.2%)
Spina bifida	1 (0.7%)
Congenital anomalies	2 (1.4%)
Primary methods of mobility	
Walking	86 (60.6%)
Walking with assistive device	22 (15.5%)
Crawling	6 (4.2%)
Carried by others	28 (19.7%)

^a^ Acquired brain injury includes traumatic brain injury, brain tumor, stroke, seizure, brain infection, brain anoxia.

**Table 3:** Difference with the CASE-C scores between children with PD and TD.

	PD	TD	ANCOVA	
	Mean (SD)	Mean (SD)	*F_(df)_*	*η_p_*^2^
Total	42.00 (8.65)	35.17 (3.19)	95.35_(1,329)_ *	0.23
Family/Community resources	43.47 (11.14)	35.22 (4.26)	81.83_(1,329)_ *	0.20
Assistance/Attitude supports	40.77 (11.28)	34.72 (3.86)	42.24_(1,329)_ *	0.11
Physical design access	41.24 (9.32)	35.50 (4.66)	57.12_(1,329)_ *	0.15

NOTES. *η_p_*^2^ = Partial eta squares (effect sizes) are reported for group differences, effect sizes are small = 0.01–0.05; medium = 0.06–0.13; large ≥ 0.14; * *p* < 0.001 based on analysis of covariances (ANCOVAs) adjusting for parental education, parental employment status, and household income category (the significance level was set at *p* < 0.0125 based on Bonferroni corrections); Abbreviations: CASE-C, the Chinese version of the Child and Adolescent Scale of Environment; PD, physical disabilities; TD, typical development.

## Abbreviations

The following abbreviations are used in this manuscript:

PD	Physical disabilities
TD	Typical development
CASE-C	Chinese version of the Child and Adolescent Scale of Environment
ICF	International Classification of Functioning, Health, and Disability
DEC	Division for Early Childhood
ANCOVA	Analysis of covariance
SD	Standard deviation
